# Bilateral iliac artery aneurysm: A rare cause of postpartum recurrent hemorrhage

**DOI:** 10.4274/tjod.galenos.2020.01336

**Published:** 2020-04-06

**Authors:** Emsal Pınar Topdağı Yılmaz, Yunus Emre Topdağı, Suat Eren, Yakup Kumtepe

**Affiliations:** 1 Atatürk University Faculty of Medicine, Department of Gynecology and Obstetrics, Erzurum, Turkey; 2 Sanko University Faculty of Medicine, Department of Gynecology and Obstetrics, Gazinatep, Turkey; 3 Atatürk University Faculty of Medicine, Department of Radiology, Erzurum, Turkey

**Keywords:** İliac artery aneurysm, postpartum hemorrhage, maternal mortality

## Abstract

Postpartum hemorrhage (PPH) is a critical health problem that may result in maternal death. In cases of impaired maternal hemodynamics, several surgical therapies such as hypogastric artery ligation or postpartum hysterectomy may be employed to control the bleeding. A 30-year-old multiparous patient who had given birth via spontaneous vaginal delivery had undergone hysterectomy and then hypogastric artery ligation due to postpartum hemorrhage. The patient was referred to our clinic due to uncontrolled bleeding and she experienced recurrent episodes of massive hemorrhage during her follow-up in our clinic. Pelvic angiography performed by interventional radiologists to detect the bleeding focus revealed arteriovenous fistula and aneurysm in the right internal iliac artery and incomplete ligation of the left internal iliac artery. The bleeding was controlled by selective embolization through coiling of the fistula in the right internal iliac artery and branches of the left uterine artery. PPH is still an important cause of maternal mortality and vascular structural anomalies must be borne in mind in cases with delayed onset.

## Introduction

Postpartum hemorrhage (PPH) is a serious health problem that may result in maternal death by causing an impairment of maternal hemodynamics^(1,2)^. According to the world health organization, approximately 150.000 women die annually of PPH^(3,4)^. Postpartum hysterectomy should be the last resort for surgeons due to its morbidity and effects on the patient’s fertility. Alternatively, hypogastric artery ligation is a fertility-preserving treatment that is associated with a low success rate and high levels of complication if not performed by experienced surgeons^(1,5)^. Pelvic arterial embolization is an alternative treatment for both hysterectomy and hypogastric artery ligation due to its effects on morbidity and patients’ fertility. It continues to be a relevant procedure because it is a minimally invasive intervention^(6,7)^. The current report presents a patient with PPH who underwent embolization, in whom bleeding could not be controlled despite repeated surgical interventions, and in whom a bleeding focus that would explain this clinical condition could not be detected intraoperatively.

## Case report

**Figure 1:**
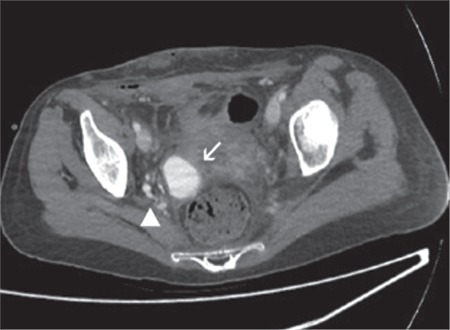
Computed tomography images acquired in the arterial phase show enlarged vascular structured connected to the arteriovenous fistula (arrow head) and aneurysm sac (white arrow)

**Figure 2:**
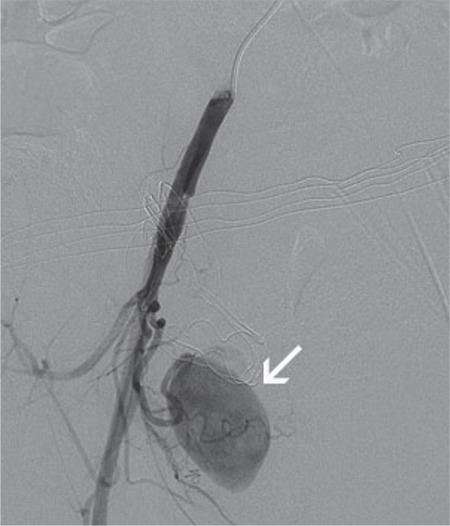
Right internal iliac artery angiography shows aneurysm sac

**Figure 3:**
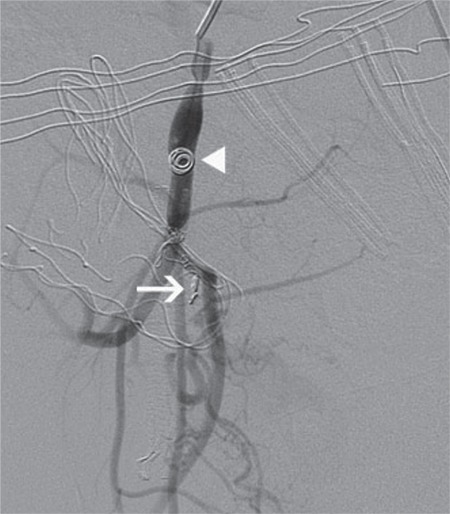
Embolization of the artery supplying the aneurysm with detachable coil (white arrow) and embolization of the internal iliac artery with pushable coil (arrow head)

The 30-year-old, gravidity 3, parity 2 patient had no medical problems during her pregnancy follow-up visits, such as gestational diabetes, preeclampsia, intrauterine growth retardation, and premature delivery. The patient had given birth to a baby weighing 3500 grams via spontaneous vaginal delivery at 39 weeks of gestation and she experienced heavy bleeding at day 6 after birth. The patient did not respond to medical therapies and conventional methods and underwent hysterectomy due to impaired hemodynamics caused by uncontrolled bleeding. The patient was discharged on postoperative day 3 with full recovery. The patient experienced active vaginal bleeding on postoperative day 10 and underwent cuff repair through the vaginal route after exploration. Possible bleeding foci that were inspected during exploration were sutured and then the patient was discharged with full recovery. However, the patient experienced recurrent abundant bleeding on postoperative day 20 for which she was hospitalized and underwent bilateral hypogastric artery ligation. The patient experienced another episode of abundant bleeding on day 7 after the hypogastric artery ligation and she was then referred to our clinic. The patient’s hemodynamics was stable on initial examination. Laboratory parameters were normal. Abdominal ultrasonography revealed normal ovaries and no fluid in the abdominal cavity. A vaginal examination revealed no bleeding. The patient experienced heavy bleeding on day 3 after admission to our clinic and she also had impairment in her hemodynamics. Her hemoglobin was 4.8 g/dL and the patient was administered 4 units of erythrocyte suspension. The patient underwent emergency surgery, but no active bleeding focus was detected. A consultation with a radiologist was performed because no bleeding focus was detected intraoperatively, and the patient underwent computed tomography (CT) with contrast enhancement. The CT scans showed findings suggestive of aneurysmal filling in the pelvic area (Figure 1). The patient had ongoing bleeding and she underwent angiography in the interventional radiology clinic. Initial angiography revealed an arteriovenous fistula and aneurysm filling from the right internal iliac artery (Figure 2). The artery fistula was closed with a coil. The right internal iliac artery was totally obstructed by the coil, causing intermittent bleeding after partial intraoperative ligation. On the second day after the intervention, the patient still had bleeding, although the amount had decreased. The patient was re-evaluated by interventional radiologists; the branches of the left uterine artery were angiographically obstructed with microparticles and the partially-ligated left internal iliac artery was totally closed by coils (Figure 3). The patient received 15 units of erythrocyte suspension until the completion of the second procedure. The patient became hemodynamically stable following the procedure and had no recurrent bleeding during the follow-up period; she was discharged with full recovery on postoperative day 15.

Informed consent was obtained from the patient.

## Discussion

PPH is defined as 500 mL blood loss after normal delivery and 1000 mL blood loss after cesarean delivery. It is a significant health problem causing an impairment in maternal hemodynamics^(1)^. If there is no response to conservative therapies such as uterine massage and Bakri balloon placement, compression sutures, hypogastric artery ligation, hysterectomy, and pelvic arterial embolization can be employed^(8,9)^.

The pelvic organs are supplied by the internal iliac artery and ligation of this artery reduces bleeding by decreasing uterine arterial pressure^(10,11)^. Pelvic arterial embolization was used for the first time in 1979 for obstetric bleeding^(7)^. Selective arterial embolization offers a success rate ranging from 81% to 96% in different sources^(12)^. Pelvic arterial embolization is considered a safe procedure because it is a fertility-preserving minimally invasive procedure and, in experienced hands, it is also associated with reduced morbidity^(1)^. However, there are also publications reporting necrosis of the uterus, sciatic nerve, and bladder if not performed under the appropriate conditions^(13,14)^.

Although uterine atony is the most common cause of PPH, vascular pathologies such as arteriovenous malformation (AVM), hemangioma, and vascular tumors must be borne in mind for patients with massive hemorrhage^(15)^. Variations in the pelvic vascular anatomy in particular may cause serious bleeding in the postpartum period. Selective arterial embolization performed after angiography is a safe and effective option in uncontrolled uterine bleeding^(2)^. There are reported cases in the literature in which selective pelvic embolization was performed due to PPH of different etiologies. Yu et al.^(15)^ reported the efficacy of selective arterial embolization in a patient with vaginal hemangioma who remained undetected in antenatal follow-up and who experienced massive bleeding after vaginal delivery with episiotomy. The development of collateral vessels between the inferior mesenteric artery (IMA) and uterus is not normally observed. However, it has been reported in patients with uterine fibroids and adenomyosis and those with a history of pelvic surgery, uterine artery ligation or embolization^(16)^. Shin et al.^(2)^ detected widespread collateral vessels between the IMA and uterine and ovarian arteries during angiography in a patient with massive PPH who had no history of uterine fibroids, pelvic surgery or embolization. They reported that the hemorrhage was controlled by the selective embolization of the distal branches of the IMA. Kim et al.^(17)^ reported a patient with massive PPH due to damage of the collateral network between the superior rectal branch of the IMA and the vaginal artery following vaginal delivery and they controlled the hemorrhage with embolization.

Vascular pathologies must be considered in patients with PPH with a delayed onset. In a series of 33 patients with PPH reported by Crespo et al.^(1)^, six patients experienced hemorrhage due to laceration of the uterine pseudoaneurysm and three patients had laceration of the branches of the vaginal, cervicovaginal and epigastric artery, and they reported the efficacy of selective embolization. In a study by Ganguli et al.^(18)^ reporting on 66 patients with PPH, a 95% success rate was reported for uterine artery embolization and the rate of complication was reported as 4.5%.

The most common causes of pseudoaneurysms occurring in the uterine artery are curettage, vaginal delivery, and genital infections^(19)^. In the study by Kiyokawa et al.^(20)^ that included six patients with cesarean scar pregnancy and uterine AVM or pseudoaneurysm, five patients underwent uterine artery embolization. Only one patient who underwent embolization resulted in hysterectomy.

The present case had intermittent hemorrhage but it was severe enough to impair hemodynamics after spontaneous vaginal delivery. A total of three operations were performed for vaginal cuff repair, hysterectomy, and finally hypogastric artery ligation because the bleeding focus could not be detected despite ongoing hemorrhage. The presence of recurrent hemorrhage and failure to detect the bleeding focus despite all plausible surgical interventions led us to consider the patient for vascular pathologies. The failure to control bleeding, after the detection of and intervention to the vascular pathology in the right side, raised the possibility of another pathology in the left side. We therefore emphasize the importance of bearing vascular pathologies in mind in patients with uncontrolled hemorrhage despite optimal surgical interventions. Consistent with the literature, we observed the successful embolization of the aneurysm accompanying the arteriovenous fistula and arterial branches causing bleeding due to incomplete suturing.

In conclusion, emergency intervention in PPH can be life-saving. Selective arterial embolization, which is less invasive and is associated with low complication rates, appears to be an appropriate option for patients unresponsive to conservative therapy.
